# The Impact of Public Opinion Pressure on Construction Company Green Innovations: The Mediating Effect of Leaders' Environmental Intention and the Moderating Effect of Environmental Regulation

**DOI:** 10.3389/fpsyg.2022.936058

**Published:** 2022-06-24

**Authors:** Bo Wang, Shan Han, Yibin Ao, Fangwei Liao, Tong Wang, Yunfeng Chen

**Affiliations:** ^1^School of Civil Engineering and Architecture, Southwest University of Science and Technology, Mianyang, China; ^2^College of Environmental and Civil Engineering, Chengdu University of Technology, Chengdu, China; ^3^School of Economics and Management, Southwest University of Science and Technology, Mianyang, China; ^4^Architecture and the Built Environment Faculty, Delft University of Technology, Delft, Netherlands; ^5^School of Construction Management Technology, Purdue Polytechnic Institute, Purdue University, Lafayette, LA, United States

**Keywords:** environmental public opinion pressure, leaders' environmental intention, incentive environmental regulation, mandatory environmental regulation, enterprise green innovation behavior

## Abstract

Media has paid more attention recently on environmental issues caused by construction companies which imposes public opinion pressure on construction companies and could potentially impact their decision-making processes for green innovations. However, research on the relationship between public opinions pressure and construction company green innovation behavior is still limited. To understand how such public opinions pressure can impact construction companies' green transition and formulate advice accordingly, it is necessary to use empirical data to find the correlations. Therefore, this research has gathered questionnaire data of the construction companies in Chengdu-Chongqing economic circle of China to study the influencing mechanism of environmental public opinion pressure on enterprise green innovation behavior, analyzes the realization path of leaders' environmental intention as a mediating variable in the impact of environmental public opinion pressure on enterprise green innovation behavior, and reveals the role boundary of environmental regulation as a moderating variable in the impact of environmental public opinion pressure on enterprise green innovation behavior. The results show that environmental public opinion pressure has a significant positive impact on enterprise green innovation behavior. More specifically, enterprise green innovation behavior is affected by leaders' environmental intention and the latter plays a partial mediating role between environmental public opinion pressure and enterprise green innovation behavior. Environmental regulation also enhances the sensitivity of companies to environmental public opinion pressure, and therefore can significantly strengthen the relationship between environmental public opinion pressure and enterprise green innovation behavior. Further research find that, compared with incentive-based environmental regulations, mandatory environmental regulations make companies more sensitive to environmental public opinion pressure and has a more significant positive moderating effect. The research conclusions could be used to provide theoretical reference with empirical data for accelerating the green innovation transformation and promoting the high-quality development in the construction industry.

## Introduction

Since the publication of “Our Common Future” drafted by Gro Harlem Brundtland in 1987, the concept of green development has spread rapidly around the world, and has become an important way to deal with a series of global problems such as climate change, strategic resource shortage and financial crisis, as well as to coordinate the contradiction between economic development and resources and environment (Yang et al., 2020, 2022). However, the ability of comprehensive environmental management is still relatively weak in developing countries (Mohsin et al., 2020). As the largest developing country in the world, China is currently in a critical period of economic structural transformation, and green development has become a new driving force for economic growth. The key of green development is green innovation and to improve environmental performance (King and Lenox, 2001). Innovation management scholars believe that innovation behavior has an important impact on green innovation action (Li et al., 2020; Luo et al., 2022). Green innovation behavior can be regarded as an important factor affecting environmental performance and green development (Li et al., 2022), which is helpful to effectively deal with various environmental problems faced in the period of economic structural transformation, and can improve environmental performance and environmental governance capabilities.

Green innovation has the dual externalities of “technology spillover effect” and “environmental cost problem,” which affects the enthusiasm of enterprises for green innovation. The existing research on the factors of enterprise green innovation behavior mainly focus on external factors and internal factors. The external factors mainly focus on stakeholder pressure (Sarkis et al., 2010), environmental regulation (Jaluza and Lara, 2019), etc. Stakeholder theory believes that stakeholders such as government, competitors, suppliers and customers will have an impact on enterprise green innovation. Wang et al. (2020) believe that government intervention affects the green innovation of enterprises. Colin and Cheng (2020) believe that the implementation of green innovation by competitors can drive enterprises to carry out green innovation actions. Horbach (2008) finds that suppliers, customers and non-governmental organizations have a positive impact on enterprise green innovations. Furthermore, institutional theory believes that the decision-making behavior of an enterprise will abide by certain rules to ensure the legality, goodwill and living space of the enterprise's production and operation. Porter and Linde (1995) concludes that is inappropriate to oppose the relationship between environmental protection and economic development, and appropriate environmental regulation can prompt enterprises to carry out more innovative activities. Berrone et al. (2013) and Meng et al. (2020) further confirm that institutional pressure and environmental regulation can significantly affect enterprise green innovation. The internal factors mainly focus on the acquisition of resource advantages (Chiarvesio et al., 2015), the core competitiveness of enterprises (Frondel et al., 2007), etc. Resource-based theory believes that enterprises have different tangible and intangible resources, these unique resources are valuable and irreplaceable, and green innovation is an important source for enterprises to obtain resource advantages (Pacheco et al., 2016). In addition, green innovation can prompt enterprises to transform current products, technologies and processes, and improve their green core competitiveness. Doran and Ryan (2016), Chen et al. (2018) believe that green innovation can help enterprises break down technical barriers and establish industry competitive advantages.

With the rapid development of modern communication technology, corporate information disclosure has become increasingly transparent, and public opinion as an effective external governance method have an important impact on corporate behavior. Enterprise environmental behavior has attracted the attention of public opinion, which forms a kind of environmental public opinion pressure on enterprises. Continued environmental public opinion pressure will cause companies to be boycotted by customers, condemned by society and punished by the government, forcing companies to pay attention to environmental issues and try their best to reduce the negative impact on the environment by adopting green innovation strategies. In the theory of institutional economics, public opinion pressure is often referred to as an informal system that affects corporate innovation (McCombs and Shaw, 1972). Green innovation is the behavioral choice of corporate innovation, and scholars have different views on whether public opinion pressure can affect enterprise green innovation. Some people believe that environmental public opinion can attract the attention of government departments and have an impact on corporate environmental governance through the administrative intervention mechanism (Dyck et al., 2008; Cu and Li, 2012). There are also some thoughts that the information disclosed by media regarding public opinions can reduce the information asymmetry between enterprises and stakeholders, and stakeholders can stop the opportunistic behavior of enterprises through market pressure (Cainelli et al., 2015), thereby affecting the green innovation decision-making of enterprises. The above viewpoints are mainly based on the perspectives of “government governance” and “market pressure” to study the impact of environmental public opinion on corporate environmental governance or green innovation, but lack of in-depth exploration of the path and mechanism of environmental public opinion pressure affecting enterprise green innovation behavior.

In fact, leaders are important decision makers in corporate strategy, leader's intention of environmental issues will affect enterprise green innovation behavior (Huang and Li, 2015). According to the Theory of Planned Behavior (TPB), factors that may affect behavior indirectly affect behavior through behavioral intentions (Ajzen, 2002), then, the green innovation behavior of enterprises may also be influenced by leaders' environmental intention. Leaders with strong environmental intention will help companies build a green culture, improve employees' awareness of green innovation, and inspire and guide enterprise green innovation actions. Therefore, environmental public opinion pressure and leaders' environmental intention are inseparable from enterprise green innovation behavior. However, incorporating the three into the same theoretical framework and exploring the mechanism that environmental public opinion pressure affects enterprise green innovation behavior still lacks theoretical and empirical support. Based on this, this paper uses the theories of agenda setting theory, theory of planned behavior, and higher-order cognitive theory to study the impact of environmental public opinion pressure on enterprise green innovation behavior, and analyzes the mediating role of leaders' environmental intention in the relationship between the two. In addition, based on the “Porter Hypothesis,” environmental regulation should be introduced as well to investigate the moderating role of environmental regulation on the influence of environmental public opinion pressure on enterprise green innovation behavior.

Therefore, the structure of the research is as follows: The first part is the theoretical review and research hypothesis. The second part is the research method, the questionnaire design and variable measurement are carried out, the questionnaire data are collected by taking the construction companies in Chengdu-Chongqing economic circle of China as the research object, the research steps and methods are explained. The third part is the empirical analysis and results, the reliability and validity of the scale data are tested, the descriptive statistics, correlation analysis and regression analysis are performed. Finally, we draw the conclusions and implications of this work. This research tries to explore the influence mechanism of environmental public opinion pressure on enterprise green innovation behavior to further broadens the research scope of enterprise green innovation behavior, and supplement the existing theoretical system. Furthermore, this research explores the realization path of leaders' environmental intention as an intermediary variable in the influence of environmental public opinion pressure on enterprise green innovation behavior and verify the relationship between environmental public opinion pressure and environmental regulation to further reveal the role boundary of environmental regulation as a moderator variable in the impact of environmental public opinion pressure on enterprise green innovation behavior.

## Theoretical Review and Research Hypotheses

### Environmental Public Opinion Pressure and Enterprise Green Innovation Behavior

Environmental public opinion pressure refers to the pressure of negative media reports, stakeholders' disclosure or reporting when enterprises have environmental governance issues (Dai and Lu, 2020). With the development of communication technology, people are accustomed to spreading news and expressing their attitudes on the Internet, positive public opinion can enhance the legitimacy of business operations and can expand its influence and market share. Negative public opinion may damage corporate image and affect corporate performance. Compared with positive information, the public is more likely to pay attention to negative information and make decisions by using negative information (Herzog and Meese, 2021). Media public opinion is also more inclined to publish negative information about enterprise environmental violations in order to attract public attention. Therefore, environmental public opinion pressure is mostly from the negative reports of the media on the environmental problems of the enterprise. In order to maintain their reputation, enterprises have to pay attention to the environmental public opinion and regard it as one of the important factors affecting the sustainable development of enterprises (Papagiannakis and Lioukas, 2012; Zhang et al., 2015; Ao et al., 2020; Yang et al., 2021).

Regarding the impact of environmental public opinion pressure on enterprise green innovation behavior, the agenda setting theory believes that corporate issue is an important part of media public opinion, media public opinion can sway people's attention to certain facts that will have an impact on the audience's attitudes or behaviors (McCombs and Shaw, 1972). Han and Cheng (2020) believe that media as important variables in activating norm perception can affect pro-environmental behavior. Media attention to environmental issues can form a “pegging effect” (Wang et al., 2022), and continuous environmental public opinion pressure can prompt enterprises to enhance their green innovation awareness, make green innovation decisions, and implement green innovation actions. After all, the continuous tracking and reporting of violations by public opinion can bring huge administrative intervention and ethical pressure to enterprises.

On the other hand, due to the high cost and high risk characteristics of green innovation, enterprises may show opportunistic behaviors in pursuit of short-term interests, becoming more “conservative” in green innovation decision-making, and even pursuing the idea of “not seeking merit, but not fault,” and then making low-risk and quick-return decisions (Colin and Cheng, 2020). This short sighted behavior will hinder the green innovation of enterprises, and environmental public opinion can attract the attention of government departments, stopping the opportunistic behavior of enterprises through administrative intervention (Cu and Li, 2012), prompting enterprises to enhance their green innovation awareness and making green innovation decisions. The legitimacy theory believes that the behavior of enterprises should conform to social morality and legal norms to gain more social recognition (Hattori, 2017; Ferris et al., 2019). In order to create a good image of green development, enterprises will tend to green innovation to gain the public's recognition and enhance their legitimacy. When enterprises have negative environmental public opinions, especially when major environmental problems occurs and enterprises are punished by government, stakeholders will express their complaints or dissatisfaction because of the company's non-green decision-making behavior, then reducing or abandoning investment, driving enterprises improve green innovation awareness, making green innovation decisions, and enhancing the legitimacy of corporate behavior. Thus, the below hypothesis is proposed.

**H1:** Environmental public opinion pressure has a significant positive impact on enterprise green innovation behavior.

### Mediating Effect of Leaders' Environmental Intention

Leaders' environmental intention refers to the subjective desire of enterprise leaders to take green innovation actions (Huang and Li, 2015). The theory of planned behavior believes that the behavior of the subject is driven by the intention (Fishbein and Ajzen, 1977). Green innovation is composed of green innovation intention and green innovation behavior, and green innovation intention is the most effective tool to predict enterprise green innovation behavior (Ajzen, 2002). The green expectations of stakeholders such as the government, suppliers, and customers do not directly affect the behavior of enterprises, but they can stimulate the green intention of enterprises to promote green innovation behaviors through the pressure of environmental governance (Li et al., 2019). Individual innovation is the beginning of organizational innovation, and enterprise green innovation behavior must ultimately be implemented into individual environmental intention (Hagger and Chatzisarantis, 2005). Higher-order cognitive theory believes that leaders' understanding of organizational environment affects corporate strategic decisions, which is determined by psychological characteristics such as leaders' individual cognition and values (Hambrick and Mason, 1984). Leaders' environmental intention is leaders' cognition and desire of the environmental protection, which is expressed in the understanding and support of environmental governance in terms of values. Begum et al. (2022) believe that environmental transformational leadership and creative process engagement positively influence green product innovation and green process innovation. Leaders with strong environmental intention can help enterprises build a green culture, improve employees' green innovation awareness, and inspire and guide companies' green innovation actions. Therefore, the stronger the leaders' environmental intention, the more likely it is to stimulate enterprise green innovation behavior.

At the same time, leaders' cognition and attitude toward environmental protection and green innovation will be affected by external environment. Theory of planned behavior believes that external environmental factors can exert some perceptible pressure on subject behavior, and then affect the individual's cognition and behavior motivation (Fishbein and Ajzen, 1977). Therefore, environmental public opinion pressure will have an impact on leaders' environmental intention. On the one hand, when enterprises have environmental problems and are exposed by public opinion, the continuous environmental public opinion pressure will cause enterprises to be boycotted by customers, condemned by the society and punished by the government, which will bring greater environmental governance pressure to enterprise leaders. Environmental public opinion pressure will enhance the green innovation cognition and subjective desire of enterprise leaders, and prompting enterprises to adopt green innovation strategies to reduce the negative impact of production and operation on the environment, which will improve the external image of the enterprise. On the other hand, enterprise leaders with strong awareness of environmental risks and environmental benefits tend to be more “active” in environmental protection (Charles et al., 2018; Myskova and Hajek, 2018). In order to avoid being exposed by public opinion, enterprises will actively respond to the country's call for green development, regarding green innovation as corporate social responsibility, and green innovation investment is regarded as a new profit growth point. Therefore, the capital investment for technological improvement is increased, environmental governance plans are formulated, and green innovation capabilities are improved.

To sum up, environmental public opinion pressure can prompt the improvement of leaders' environment intention, and making enterprise leaders to realize the importance of green innovation to the sustainable development of enterprises. Leaders are important decision makers of enterprises, and their cognition and attitude toward green innovation will determine the direction of enterprise green innovation behavior. Thus, the below hypothesis is proposed.

**H2a:** Environmental public opinion pressure has a significant positive impact on Leaders' environmental intention.

**H2b:** Leaders' environmental intention has a significant positive impact on enterprise green innovation behavior.

**H2c:** Leaders' environmental intention has a mediating effect on the relationship between environmental public opinion pressure and enterprise green innovation behavior.

### Moderating Effect of Environmental Regulation

Environmental regulation refers to the regulation of various behaviors that pollute the public environment (Zhou et al., 2021). As the ecological binding force of resources, its main purpose is to achieve a balance between economic growth and environmental protection (Xu et al., 2022). Through the institutional constraints on the behavior of enterprises, the environmental quality can be improved and the sustainable development of the economy can be promoted. The research on the impact of environmental regulation on enterprise green innovation can be traced back to the “Porter Hypothesis.” Porter and Linde (1995) believe that appropriate environmental regulation can encourage enterprises to carry out more innovation activities, and these innovations will improve the productivity of enterprises, offsetting the costs caused by environmental protection and improving the profitability of enterprises in the market. Since green innovation has the dual externalities of “technology spillover effect” and “environmental cost problem,” enterprises are less motivated to actively implement green innovation. Environmental regulation can prompt enterprises to continuously improve their internal management, urging enterprises to pay attention to green technology and environment-friendly product innovation, and encouraging enterprises to increase investment in green research and development, which will ultimately help improve their green innovation performance. Xu et al. (2022) believe that environmental regulations positively affect green innovation through short-term or long-term external financing. Richard and Edward (2011); Ford et al. (2014); Peng et al. (2021), and Paramati et al. (2022) have also confirmed that the government can effectively promote enterprises to implement green innovation actions through environmental regulation studied by different regions and scenarios.

Environmental regulation can be divided into incentive environmental regulation and mandatory environmental regulation. Incentive environmental regulation refers to the government's incentives to enterprises through economic means such as R&D subsidies, innovation rewards, and tax relief. Mandatory environmental regulation refers to the government imposes mandatory policy constraints on enterprise behavior in the form of laws and regulations. Institutional theory believes that enterprises have the characteristics of social embeddedness and external control (Martin, 1990). Due to different political systems in different countries, there are great differences in environmental regulations, the effect of environmental regulation varies regionally especially in China (Xu et al., 2022). In the development process of emerging market countries, the government has controlled the key resources required by enterprises, and uses macro-control and market supervision to allocate resources, at the same time, the government needs multi-party supervision to improve environmental regulations. Xu et al. (2022) focused the research sample on Chinese A-share polluting companies and believe that there are differences in the impact of environmental regulation on enterprise green innovation in different regions, industries, and property rights in China. The public opinion governance mechanism is very dependent on the government governance (Etter et al., 2018). China is in a period of economic restructuring and transformation, the public opinion supervision mechanism is not perfect especially, and factors such as environmental regulation will greatly affect government governance function of public opinion. Environmental public opinion pressure brought by media will affect the implementation of government environmental regulations, increase the probability of government intervention in violating enterprises, and to some extent, promote the environmental governance function of media public opinion.

Therefore, under the pressure of environmental public opinion, the stronger the incentive environmental regulation is, the more tax deductions and financial rewards enterprises can obtain from the government, and the risk of green innovation will be reduced, which will help enterprises to be more concerned about the environment, strengthening enterprises green environmental awareness, and stimulating green innovation behaviors. Samely, the stronger the mandatory environmental regulation is, the greater the government's environmental protection and punishment is, and the higher the cost of companies' violations are. Under the pressure of environmental public opinion, companies face legality issues such as administrative intervention and ethics. Legitimacy theory believes that enterprises can improve government satisfaction by increasing investment in green innovation, then obtaining the status of “legitimacy,” which can help companies obtain preferential policies and market competitive advantages (Song and Yu, 2018). Once corporate environmental violations are exposed by the media, companies are more likely to attract government attention and receive administrative penalties, greatly reducing government satisfaction and weakening their market competitiveness. In order to restore market reputation and improve government satisfaction, enterprises will strengthen their intentions to innovate and implement green innovation actions. Therefore, environmental regulation can enhance the sensitivity of enterprises to environmental public opinion pressure, which will enable environmental public opinion to play a better role in the supervision and governance of enterprises green innovation. Thus, the below hypotheses are proposed.

**H3a:** Incentive environmental regulation has a positive moderating effect on the relationship between environmental public opinion pressure and enterprise green innovation behavior.

**H3b:** Mandatory environmental regulation has a positive moderating effect on the relationship between environmental public opinion pressure and enterprise green innovation behavior.

For enterprises, due to the different sources of pressure and legitimacy generated by mandatory environmental regulation and incentive environmental regulation, there may be differences in their influence mechanisms on enterprise green innovation behavior (Li et al., 2017). For mandatory environmental regulation, what it imposes is a legal mechanism of coercive significance, which can affect the behavior of the organization through the punishment mechanism, especially when enterprises violate the rules and are exposed by public opinion. This kind of coercive pressure has a far-reaching impact on enterprises. Under the pressure of environmental public opinion, mandatory environmental regulation can accelerate the promotion of green innovation-related issues into the decision-making process of enterprises, forcing enterprises to increase resource investment in green innovation. Correspondingly, incentive environmental regulation mainly affects organizational behavior by affecting the allocation of corporate resources or incentive mechanism (Fischer et al., 2003). Companies are relatively less sensitive to environmental public opinion pressures, and will have reservations in corporate decision-making and green innovation resource investment. Thus, the below hypothesis is proposed.

**H3c:** Compared with incentive environmental regulation, mandatory environmental regulation has a stronger positive moderating effect on the relationship between environmental public opinion pressure and enterprise green innovation behavior.

**Figure 1:**
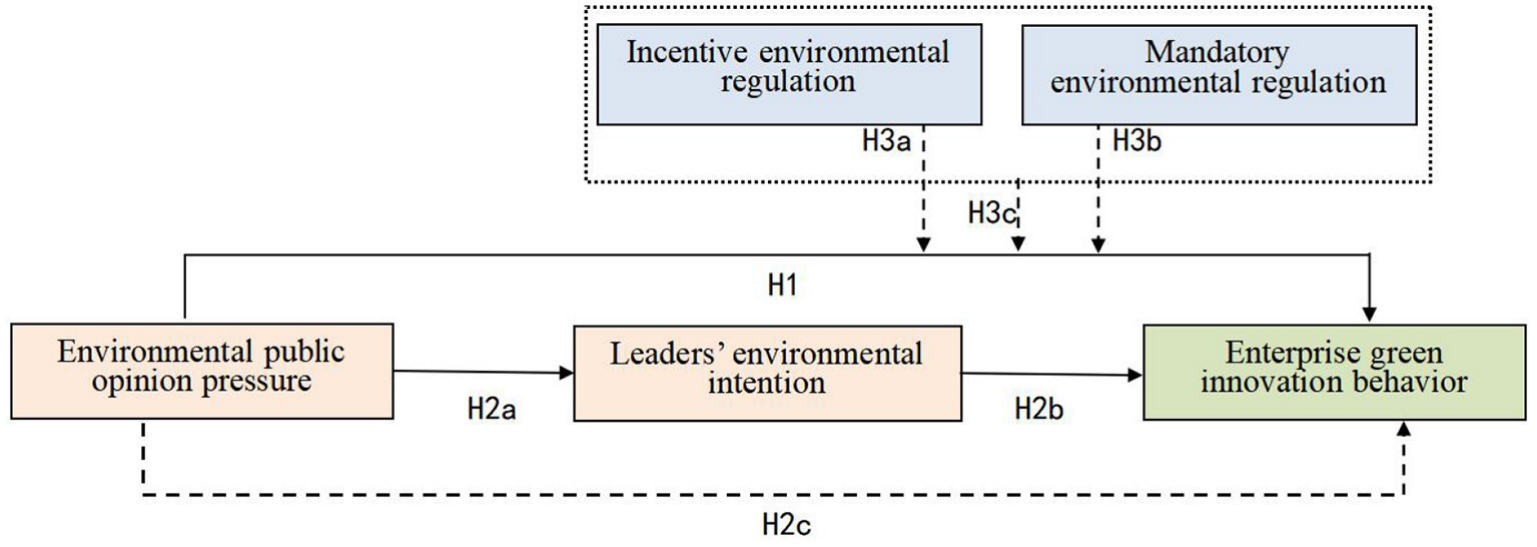
Theoretical model.

The theoretical model of the research is shown in Figure 1.

## Research Methods

### Questionnaire Design and Measurement of Variable

Questionnaire for this research used a Likert 5-point scale (1 = “strongly disagree,” 5 = “strongly agree”). Firstly, we referred to the relatively mature scales in the classic literature to form a preliminary questionnaire to ensure the reliability and validity of the variable measurement. Secondly, the semantic structure and content of the questionnaire items were determined, and a formal questionnaire was formed under the discussion of the members of the research group. Finally, in order to ensure that the questionnaire can accurately describe the variables involved in the research, we conducted exchanges and interviews with some enterprise leaders to better grasp the characteristics of the variables in the study, which could help modify the relevant items and make the design of the scale items more reasonable and pertinent. Then, we carried out pre-tests to ensure the validity of the questionnaires.

(1) Enterprise Green Innovation Behavior (EGIB)
  Regarding the green innovation behavior scale, this paper referred to the research of Li et al. (2019), highlighted the characteristics of enterprise green innovation behavior in content, and designed the scale from four aspects: enterprise green innovation planning behavior, material procurement behavior, research and development behavior, and construction and operation behavior. A total of 4 items were set, and the higher the score of each item is, the stronger the enterprise green innovation behavior is.
(2) Environmental public opinion pressure (EPOP)
  There are relatively few studies on environmental public opinion pressure. Since Dyck et al. (2008) confirmed the role of media in corporate governance, it has mainly been measured around the number of media reports, but the measurement method has the limitation of large data errors. In terms of scale design, Zhang et al. (2015) compiled an institutional pressure scale. Dai and Lu (2020) compiled an public opinion pressure measurement scale combined with the characteristics of public opinion. Therefore, this paper referred to the public opinion pressure scale compiled by Dai and Lu (2020), and combined with the characteristics of environmental public opinion pressure. The scale was designed from three aspects: environmental public opinion regulation pressure, environmental public opinion normative pressure and environmental public opinion imitation pressure, and a total of 4 items were set. And the higher the score of each item is, the greater the environmental public opinion pressure faced by the enterprise is.
(3) Leaders' Environmental Intention (LEI)
  Fishbein and Ajzen (1977) believes that the behavior of the subject is driven by the intention. Higher-order cognitive theory believes that leaders' understanding of organizational environment is determined by psychological characteristics such as leaders' individual cognition and values (Hambrick and Mason, 1984). Begum et al. (2022) believe that environmental transformational leadership and creative process engagement positively influence green innovation behavior. In terms of scale design of leaders' environmental intentions, Gadenne et al. (2009) measured executive environmental awareness based on their environmental attitudes, aspirations and decision-making. Therefore, this research referred to the executive environmental awareness scale compiled by Gadenne et al. (2009), combined with the connotation of individual green innovation intention, the scale of leaders' environmental intention was designed from three aspects: leaders' environmental awareness, leaders' environmental aspirations, and leaders' environmental decision-making.
(4) Environmental Regulation (ER)
  Environmental regulation was divided into two aspects: Incentive environmental regulation (IER) and mandatory environmental regulation (MER). In scale design, Ford et al. (2014), Xing and Yu (2020) designed incentive environmental regulation scale from capital subsidies, tax incentives and corporate loans. Ford et al. (2014), Cao and Sun (2021) designed mandatory environmental regulation scale from laws and regulations, environmental policies, environmental supervision and enterprise standards. Therefore, this paper referred to the research of Ford et al. (2014), Cao and Sun (2021), etc., the scale of incentive environmental regulation was designed from three aspects: capital subsidies, tax incentives, and corporate loans. The scale of mandatory environmental regulation was designed from four aspects: laws and regulations, environmental policies, environmental supervision and enterprise standards.
(5) Control Variables
  Referring to existing research and combining with previous research interviews, the enterprise background may have an impact on green innovation behavior. Therefore, this research selected the enterprise age (AGE), enterprise size (SIZE), property rights (STATE) as control variables. The age of the enterprise was measured by the difference between the year of the questionnaire collection (2021) and the year when the enterprise was established, then processed by natural logarithm. The natural logarithm of the number of employees in an enterprise was used as the measure of enterprise size. For property rights, the sample enterprises were divided into state-owned group and non-state-owned group, and state-owned enterprises were assigned with a value of “1” and non-state-owned enterprises were assigned with a value of “0.”

### Sample Selection and Data Collection

This paper selected Chinese construction enterprises as the empirical object. On the one hand, the construction industry is the lifeblood of the national economy. In China, it has made important contributions in promoting economic growth, relieving employment pressure and improving people's quality of life. At the same time, the construction industry is also facing very serious problems such as high energy consumption, high carbon emissions and high environmental pollution. In particular, the construction industry is prone to many environmental governance problems at different stages such as building materials production, building construction and operations (Liu and Dong, 2019), which often become the focus of media public opinion. At the same time, the construction market, as an important part of China's super-large-scale market, is an important position for China to build a new development pattern. Therefore, accelerating the development of green innovation in construction industry is of great significance to improving China's environmental governance and achieving China's “dual carbon” goal.

(1) Sample Selection
  This paper takes the construction enterprises in Chengdu-Chongqing economic circle of China as the research object. Firstly, it takes the “High quality living place” as one of the strategic orientations in “Outline of the Construction Planning of Chengdu-Chongqing Economic Circle,” and the construction industry is an important carrier to build a “High quality living place.” More importantly, Chengdu-Chongqing economic circle is the urbanization area with the highest level of development in western China. The green innovation development of Chengdu-Chongqing region has important radiation and driving significance for the high-quality economic development of western China.
(2) Data Collection
  We collected data in the form of questionnaires. In order to ensure the quality of the data, a threshold was set for the respondents of the questionnaires. We required the respondents to be middle or senior managers or key technical personnel who have a better understanding of corporate strategy and innovation.

**Figure 2:**
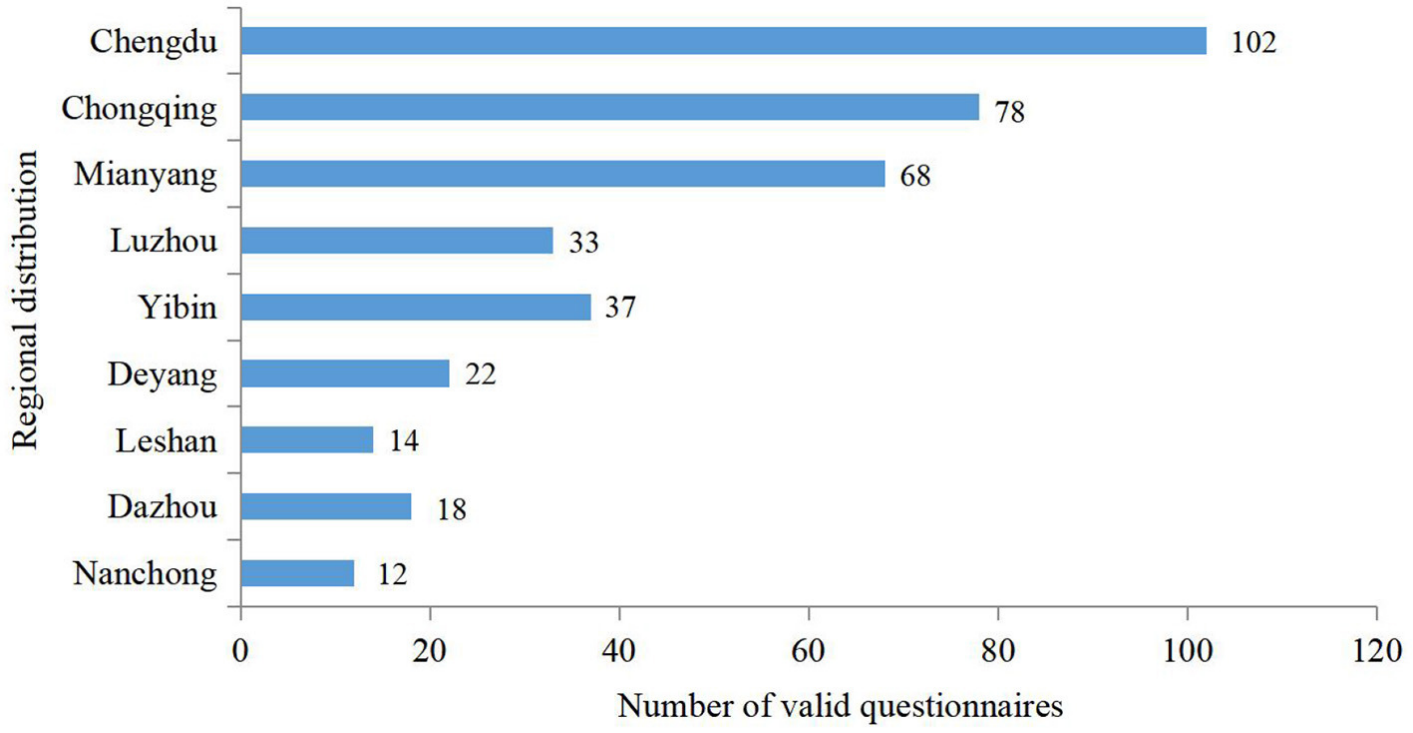
Regional distribution of valid questionnaires.

To ensure the reliability and validity of the scale, the data collection of this research was divided into two stages. The first stage was pre-testing, which began in June 2021 and ended in July 2021, we took professors, associate professors, doctors, and corporate alumni in the relevant fields of the author's university as the survey respondents, a total of 80 questionnaires were distributed, and 72 valid questionnaires were obtained. We used SPSS 22.0 software to conduct independent samples *T*-test, and the results showed that all items had good discrimination. The reliability analysis of the scale showed that the Cronbach α value of all variables were >0.700, which indicated that the design of the questionnaire had good reliability. Exploratory factor analysis was performed on all items, and the factor loadings of the rotating component matrix were all >0.600, which was in line with the expectations of the questionnaire design, and the questionnaire could be formally distributed. The second stage was the formal test, which began in September 2021 and ended in November 2021. Questionnaires were distributed to construction companies in the Chengdu-Chongqing economic circle (including Chongqing, Chengdu, Mianyang, Deyang, Yibin, Luzhou, Leshan, Dazhou, and Nanchong). With the help of college teachers, MBA students and corporate alumni, questionnaires were distributed by filling in on-site and by emails. We distributed questionnaires to 523 companies, 412 questionnaires were returned, and 384 valid questionnaires were obtained. The rate of valid questionnaires was 73.4%. As is shown in Figure 2, Chengdu, Chongqing and Mianyang are the main regions with valid questionnaires, the number of valid questionnaires in the above three regions accounted for 64.6% of the total.

### Research Steps and Methods

This research took the following steps and methods to test the scale data and research hypotheses. Firstly, common method bias test referencing Zhou and Long (2004) was applied, and Harman's single-factor test to perform an un-rotated factor analysis on all the collected questionnaire data to test the common method bias was used. Secondly, reliability and validity analysis was performed. Reliability analysis is a method to measure the reliability of the scale, and validity analysis is a method to measure the accuracy of the scale. This research used SPSS 22.0 software and AMOS 22.0 software to test the reliability of the scale, then test the validity of the scale through exploratory factor analysis, confirmatory factor analysis and model fit analysis. Thirdly, descriptive statistics and correlation analysis was performed. We performed descriptive statistical analysis on the sample data, used the Pearson correlation coefficient to test the relationship between the variables, and used the variance inflation factor (VIF) to test whether there is a collinearity problem between the variables. Fourthly, regression analysis and hypothesis testing was executed. This research followed the four-step regression analysis method proposed by Baron and Kenny (1986) to test the main effect and mediating effect. We followed the moderating effect test procedure proposed by Wen and Ye (2014) to test the moderating effect. Finally, we used Bootstrapping and other methods to test the robustness of the research hypotheses.

## Empirical Analysis and Results

### Common Method Bias Test

In the process of filling in the questionnaire, we have taken measures such as anonymity and confidentiality, but common method bias test is still required for the questionnaire. Therefore, we used SPSS 22.0 software to test common method bias, and the results showed that the unrotated first principal factor explained 38.77% of the variance variation, which did not exceed 40% of the total variance, indicating that the common method bias was not serious and the next step could be performed.

### Reliability and Validity Analysis

As shown in Table 1, the Cronbach α coefficient was used to test the reliability of the scale. The Cronbach α values of all variables were >0.700, indicating that the scale had high reliability and good stability and internal consistency. Exploratory factor analysis was performed on all items, and the KMO value was 0.809, which was >0.600. The significance of Barlett's test was 0.000. The total explanation rate of variance for each factor was 80.883%, which was >60%, showing that it was suitable for factor analysis and can be carried out. The scale used in this research refers to the existing literature, and has been verified and pre-tested by experts to ensure its content validity. Through confirmatory factor analysis, the factor loading of most indicators of the scale were >0.600; the average variance extracted value of each variable (AVE) was >0.500; and the combined reliability (CR) was >0.700. As shown in Table 2, the square roots of AVE on the diagonal were all larger than the correlation coefficient values of this variable and other variables. At the same time, the fit of the measurement model was analyzed, and the results showed that χ2/df = 4.572, <5; RMSEA = 0.097, SRMR = 0.052, all <0.10; RMR = 0.036, <0.05; CFI = 0.936, NFI = 0.920, TLI = 0.916, IFI = 0.937, all >0.9, indicating that the scale has good discriminant validity.

**Table 1:** Reliability and validity test.

**Variable**	**Items**	**Cranach's alpha**	**Factor loadings**	**AVE**	**CR**
Environmental public opinion pressure (EPOP)	EPOP1	0.828	0.985	0.530	0.809
	EPOP2		0.643		
	EPOP3		0.416		
	EPOP4		0.830		
Incentive environmental regulation (IER)	IER1	0.849	0.795	0.599	0.853
	IER2		0.896		
	IER3		0.691		
	IER4		0.655		
Mandatory environmental regulation (MER)	MER1	0.764	0.864	0.501	0.791
	MER2		0.480		
	MER3		0.740		
	MER4		0.816		
Leaders' environmental intention (LEI)	LEI1	0.886	0.643	0.669	0.889
	LEI2		0.851		
	LEI3		0.856		
	LEI4		0.921		
Enterprise green innovation behavior (EGIB)	EGIB1	0.902	0.888	0.797	0.934
	EGIB2		0.938		
	EGIB3		0.534		
	EGIB4		0.963		

**Table 2:** Descriptive statistics and correlation analysis (*N* = 384).

**Variable**	**1**	**2**	**3**	**4**	**5**	**6**	**7**	**8**
SIZE	N/A							
STATE	0.261^***^	N/A						
AGE	−0.009	0.111*	N/A					
EPOP	0.192^***^	0.117^*^	0.093	0.728				
IER	0.152^**^	0.135^**^	0.150^**^	0.119^*^	0.774			
MER	0.245^***^	0.135^**^	0.263^***^	0.223^***^	0.661^***^	0.708		
LEI	0.209^***^	0.167^**^	0.203^***^	0.299^***^	0.250^***^	0.627^***^	0.818	
EGIB	0.303^***^	0.213^***^	0.308^***^	0.344^***^	0.457^***^	0.622^***^	0.631^***^	0.893
Mean	2.889	0.43	1.108	2.822	3.999	4.170	3.744	4.223
Std. Dev	0.711	0.496	0.089	0.581	0.698	0.654	0.829	0.661
VIF	1.160	1.111	1.097	1.124	1.949	3.102	1.160	N/A

***, **, and * *indicate p < 0.001, p < 0.01, p < 0.05, respectively. The diagonal data is the square root of the variable AVE*.

### Descriptive Statistics and Correlation Analysis

As shown in Table 2, environmental public opinion pressure was significantly positively correlated with leaders' environmental intention (γ = 0.299, *p* < 0.001), which was significantly positively correlated with enterprise green innovation behavior (γ = 0.344, *p* < 0.001). Leaders' environmental intention was significantly positively correlated with enterprise green innovation behavior (γ = 0.631, *p* < 0.001). Incentive environmental regulation was significantly positively correlated with enterprise green innovation behavior (γ = 0.457, *p* < 0.001). Mandatory environmental regulation was significantly positively correlated with enterprise green innovation behavior (γ = 0.622, *p* < 0.001). Company size was significantly positively correlated with enterprise green innovation behavior (γ = 0.303, *p* < 0.001). Company state was significantly positively correlated with enterprise green innovation behavior (γ = 0.213, *p* < 0.001). Company age was significantly positively correlated with enterprise green innovation behavior (γ = 0.308, *p* < 0.001). In addition, the multicollinearity problem was tested by calculating the VIF value, and the VIF coefficients of each variable were all lower than the critical value of 10, indicating that there is no multicollinearity problem in the research data.

### Main and Mediating Effect Test

As shown in Table 3, The results of model ① shows that the regression coefficient of environmental public opinion pressure was 0.263, *p* < 0.001, indicating that environmental public opinion pressure has a significant positive impact on enterprise green innovation behavior of enterprises. This is because the continuous pressure of environmental public opinion will make enterprises face the pressure of administrative intervention, ethics and legitimacy, prompting enterprises to increase their awareness of green innovation and make green innovation decisions and actions. Thus, H1 is verified.

**Table 3:** Results of main and mediating effect test.

**Variable**	**EGIB**	**LEI**	**EGIB**	**EGIB**
	**Model①**	**Model②**	**Model③**	**Model④**
EPOP	0.263^***^	0.247^***^		0.137^**^
LEI			0.546^***^	0.511^***^
SIZE	0.231^***^	0.142^**^	0.176^***^	0.159^***^
STATE	0.091^*^	0.081	0.055	0.050
AGE	0.276^***^	0.172^***^	0.193^***^	0.188^***^
*R* ^2^	0.265	0.152	0.469	0.486
Δ*R*^2^	0.257	0.143	0.464	0.479
F	34.144	16.989	83.775	71.477

***, **, and * *indicate p < 0.001, p < 0.01, p < 0.05, respectively*.

To test the mediating effect of leaders' environmental intention, firstly, we have already tested the influence of environmental public opinion pressure on enterprise green innovation behavior of enterprises, and the results of model ① have been confirmed. Secondly, we examined the influence of environmental public opinion pressure on leaders' environmental intention, and the results of model ② showed that the regression coefficient of environmental public opinion pressure was 0.247, *p* < 0.001, indicating that environmental public opinion pressure has a significant positive impact on leaders' environmental intention. Thirdly, we examined the influence of leaders' environmental intention on enterprise green innovation behavior, and the results of model ③ showed that the regression coefficient of leaders' environmental intention was 0.546, *p* < 0.001, meaning that leaders' environmental intention has a significant positive impact on enterprise green innovation behavior. Fourthly, the model was constructed by introducing mediator variables on the basis of independent variables, and the results of model 4 showed that the regression coefficient of environmental public opinion pressure was 0.137, *p* < 0.001, and the regression coefficient of leaders' environmental intention was 0.511, *p* < 0.001. At the same time, since the regression coefficient of environmental public opinion pressure in model ④ was smaller than that in model 1, which indicates that leaders' environmental intention plays a partial mediating role between environmental public opinion pressure and enterprise green innovation behavior. Leaders' intention toward green innovation determine the direction of corporate green innovation behavior, and environmental public opinion pressure can enhance corporate leaders' subjective desire for green innovation, prompting companies to take green innovation actions to improve the company's external image. Thus, H2a, H2b, H2c are verified.

### Moderating Effect Test

Table 4 gives the results of the moderation effect test. The results of model⑤ showed that the regression coefficient of the interaction term of environmental public opinion pressure and incentive environmental regulation was 0.190, *p* < 0.01, indicating that the interaction term of environmental public opinion pressure and incentive environmental regulation has a significant positive impact on enterprise green innovation behavior, suggesting that incentive environmental regulation has a positive moderating effect on the relationship between environmental public opinion pressure and enterprise green innovation behavior. H3a is verified. The results of model ⑥ showed that the regression coefficient of the interaction term between environmental public opinion pressure and mandatory environmental regulation was 0.230, *p* < 0.001, implicating that the interaction term between environmental public opinion pressure and mandatory environmental regulation has a significant positive impact on enterprise green innovation behavior, indicating that mandatory environmental regulation has a positive moderating effect on the relationship between environmental public opinion pressure and enterprise green innovation behavior. H3b is verified. At the same time, by observing the significance level of the interaction term coefficient in model ⑤ and model⑥, the interaction term of environmental public opinion pressure and mandatory environmental regulation has a more significant impact on enterprise green innovation behavior, indicating that compared with incentive environmental regulation, mandatory environmental regulation has a stronger positive moderating effect on the relationship between environmental public opinion pressure and enterprise green innovation behavior. This is because under the mandatory environmental regulation, enterprises are more sensitive to environmental public opinion pressure, and the supervision and governance function of environmental public opinion on enterprise green innovation behavior is easier to play. Thus, H3c is validated.

**Table 4:** Results of moderating effect test.

**Variable**	**EGIB**		
	**Model①**	**Model⑤**	**Model⑥**
EPOP	0.263^***^	0.222^***^	0.166^***^
IER		0.210^**^	
MER			0.332^***^
EPOP × IER		0.190^**^	
EPOP × MER			0.230^***^
SIZE	0.231^***^	0.183^***^	0.119^**^
STATE	0.091^*^	0.065	0.068
AGE	0.276^***^	0.233^***^	0.144^***^
*R* ^2^	0.265	0.399	0.497
Δ*R*^2^	0.257	0.389	0.489
F	34.144	41.690	61.982

***, **, and * *indicate p < 0.001, p < 0.01, p < 0.05, respectively*.

### Robustness Test

The Bootstrap method (sample size is 5,000, confidence interval is 95%) was used to test the robustness of the main effect of environmental public opinion pressure and the mediating effect of leaders' environmental intention and the moderating effect of environmental regulation. As shown in Table 5, the effect value of environmental public opinion pressure on enterprise green innovation behavior was 0.299, and the confidence interval was [0.194, 0.401], excluding 0. The effect value of environmental public opinion pressure on leaders' environmental intention was 0.352, and the confidence interval was [0.203, 0.492], excluding 0. Adding leaders' environmental intention as an independent variable, the effect value of environmental public opinion pressure was 0.156, with the confidence interval was [0.074, 0.241], excluding 0. The effect value of leaders' environmental intention was 0.407, and the confidence interval was [0.347, 0.464], excluding 0. The results further showed that the environmental public opinion pressure can significantly and positively affect enterprise green innovation behavior, and leaders' environmental intention has a mediating effect on the relationship between environmental public opinion pressure and enterprise green innovation behavior.

**Table 5:** Bootstrapping tests of mediating effect and moderating effect.

**Variable**	**Effect**	**Standard**	**Bootstrapping**	
**relationship**	**value**	**deviation**	**95% CI**	
			**LLCI**	**ULCI**
EPOP → EGIB	0.299	0.053	0.194	0.401
EPOP → LEI	0.352	0.047	0.203	0.492
EPOP → LEI → EGIB	0.156	0.042	0.074	0.241
EPOP × IER → EGIB	0.251	0.088	0.075	0.419
EPOP × MER → EGIB	0.304	0.083	0.141	0.466

The moderating effect value of incentive environmental regulation and environmental public opinion pressure was 0.251, and the confidence interval was [0.075, 0.419], excluding 0. The moderating effect value of mandatory environmental regulation and environmental public opinion pressure was 0.304, and the confidence interval was [0.141, 0.466], excluding 0. This showed that both incentive environmental regulation and mandatory environmental regulation can significantly strengthen the relationship between environmental public opinion pressure and enterprise green innovation behavior.

**Figure 3:**
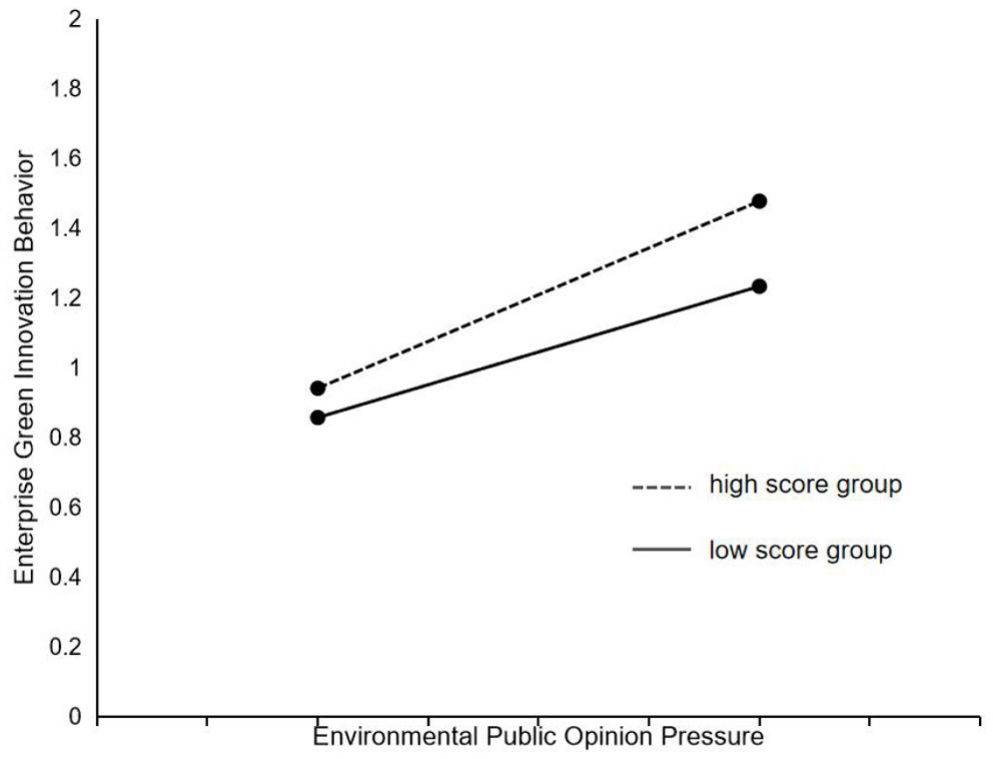
The moderating effect of incentive environmental regulation.

**Figure 4:**
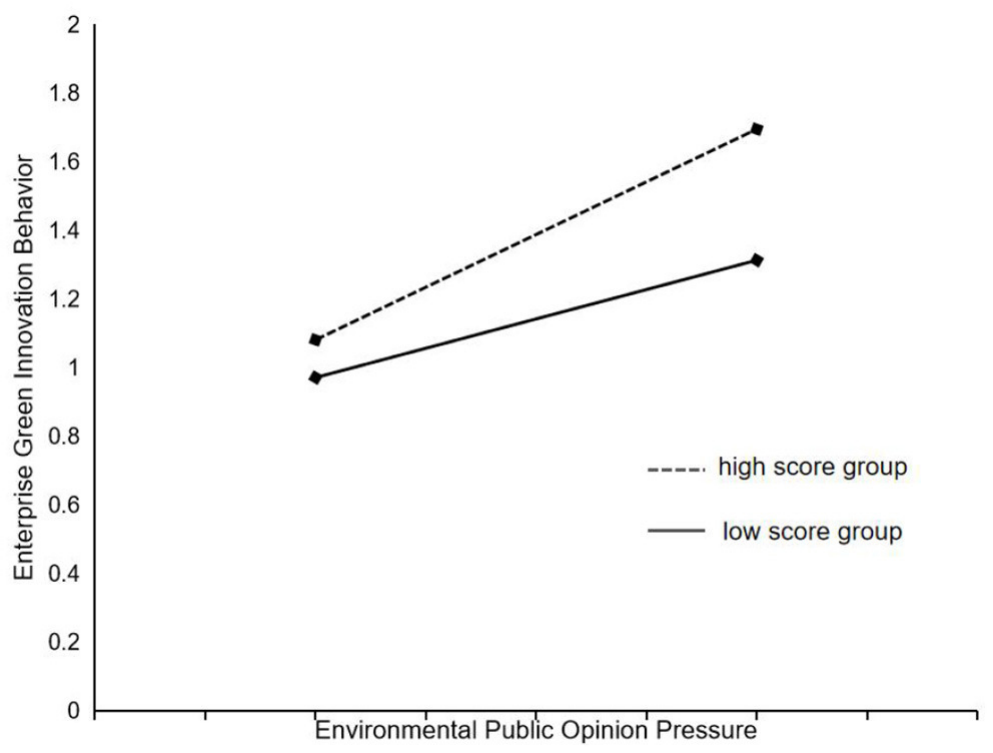
The moderating effect of mandatory environmental regulation.

In order to intuitively confirm the moderating effect of environmental regulation between environmental public opinion pressure and enterprise green innovation behavior, this research used the method of Toothaker (1994) by adding and subtracting one standard deviation from the mean of the indicators of incentive environmental regulation and mandatory environmental regulation, respectively, to divide them into high score group and low score group. The plotting the moderating effect maps are shown in Figures 3, 4. The results showed that the slopes of the high and low score groups were all positive, and the slope of high score group was slightly larger than that of low score group, indicating that they both can significantly positively strengthen the relationship between environmental public opinion pressure and enterprise green innovation behavior, and the higher the level of environmental regulation is, the stronger the moderating effect of environmental regulation is.

According to the heterogeneity of property rights of enterprises, we divided the research samples into state-owned enterprise group and non-state-owned enterprise group, and repeated the moderating effect test. As shown in Table 6, from the results of Model ⑦, Model ⑧, Model ⑨, and Model ⑩, it can be seen that whether in the state-owned enterprise group or in the non-state-owned enterprise group, compared with incentive environmental regulation, the interaction term between mandatory environmental regulation and environmental public opinion pressure has a higher level of significance, which further shows that the positive moderating effect of mandatory environmental regulation on the relationship between environmental public opinion pressure and enterprise green innovation behavior is stronger.

**Table 6:** Equity heterogeneity test of moderating effects.

**Variable**	**EGIB**			
	**State-owned**		**Non-state-owned**	
	**enterprise group**		**enterprise group**	
	**Model⑦**	**Model⑧**	**Model⑨**	**Model⑩**
EPOP	0.269^***^	0.216^***^	0.193^**^	0.136^*^
IER	0.185		0.216^*^	
MER		0.298^***^		0.341^***^
EPOP × IER	0.157		0.197^*^	
EPOP × MER		0.176^*^		0.252^**^
SIZE	0.328^***^	0.242^***^	0.055	0.028
STATE				
AGE	0.200^**^	0.160^**^	0.279^***^	0.151^**^
*R* ^2^	0.501	0.553	0.308	0.433
Δ*R*^2^	0.485	0.538	0.292	0.420
F	31.876	39.264	18.940	32.524

***, **, and * *indicate p < 0.001, p < 0.01, p < 0.05, respectively*.

## Conclusions and Implications

### Conclusion and Discussion

As the world's largest developing country, China has elevated green development to the national strategy, and green innovation is becoming a new driving force for China's economic growth. Based on 384 valid questionnaires of China, this research empirically tested the influence mechanism of environmental public opinion pressure on enterprise green innovation behavior, analyzed the realization path of leaders' environmental intention as an mediating variable in the impact of environmental public opinion pressure on enterprise green innovation behavior, and revealed the role boundary of environmental regulation as a moderating variable in the impact of environmental public opinion pressure on enterprise green innovation behavior. The main conclusions obtained are as follows.

Firstly, environmental public opinion pressure has a significant positive impact on enterprise green innovation behavior. Since Dyck et al. (2008), Cu and Li (2012) confirmed the role of media public opinion in corporate governance, academia has carried out extensive research on the impact of media public opinion on corporate decision-making and behavior. Media public opinion is an important channel for stakeholders to understand the production and operation of the enterprise. When enterprises encounter environmental problems, it will face environmental public opinion pressure from media reports, stakeholders' disclosure or reporting. Social condemnation and government punishment have forced companies to take environmental issues seriously. This research confirmed the impact of environmental public opinion pressure on enterprise green innovation behavior, and environmental public opinion pressure will prompt enterprises to improve their awareness of green innovation, make green innovation decisions, and implement green innovation actions.

Secondly, leaders' environmental intention has a mediating effect on the relationship between environmental public opinion pressure and enterprise green innovation behavior. Enterprise green innovation behavior is driven by leaders' environmental intention, and leaders' understanding and cognition of organizational environment determine enterprise green innovation behavior. This research not only confirmed the role of corporate leaders' values, leaders' awareness on enterprise green innovation (Myskova and Hajek, 2018), but also further verified the path that environmental public opinion pressure affects enterprise green innovation behavior. Under the influence of environmental public opinion pressure, leaders are more willing to implement green innovation, and then actively build an enterprise green innovation culture, improve employees' awareness of green innovation, and guide companies to carry out green innovation actions.

Thirdly, both incentive environmental regulation and mandatory environmental regulation have a positive moderating effect on the relationship between environmental public opinion pressure and enterprise green innovation behavior. Etter et al. (2018) believes that public opinion pressure brought by media would affect the implementation of government environmental regulations, increasing the probability of government intervention of companies that violate regulations, and to some extent, promoting the environmental governance function of media public opinion. However, the existing studies still lack empirical tests whether there are differences in the relationship between environmental public opinion and enterprise green innovation behavior for different types of environmental regulations. This research found that environmental regulation can enhance the sensitivity of enterprises to environmental public opinion pressure. Both incentive environmental regulation and mandatory environmental regulation have a positive moderating effect on the relationship between environmental public opinion pressure and enterprise green innovation behavior. At the same time, there are differences in the moderating effect, under the mandatory environmental regulation, enterprises are more sensitive to environmental public opinion pressure, and the supervision and governance function of environmental public opinion on enterprise green innovation behavior is easier to play.

### Theoretical Contributions and Management Implications

The theoretical contributions of this paper are as follows.

Firstly, the existing research didn't give a clear answer to whether environmental public opinion pressure can affect enterprise green innovation behavior. This paper verified the influence mechanism of environmental public opinion pressure on enterprises green innovation behavior. Under the pressure of environmental public opinion, enterprises often take green innovation actions to improve environmental governance in order to maintain their public image, enhance their competitiveness and sustainable development ability. The research results not only confirmed the viewpoint of agenda setting theory, but also can provide a new perspective for the study of green innovation behavior.

Secondly, environmental public opinion pressure can affect enterprise leaders' perception of green innovation, prompting enterprise leaders to enhance their awareness and their intention of green innovation, and reducing the negative impact of production and operation on the environment by taking green innovation actions. The research results can confirm the viewpoints of planned behavior theory and higher-order cognitive theory, and make up for the lack of attention paid to leaders' cognitive level of enterprise in green innovation research pointed by Lewis et al. (2014). At the same time, it also can enrich the research on the influencing factors of enterprise green innovation behavior theoretically, and reveal the influence path of environmental public opinion pressure on enterprise green innovation behavior.

Thirdly, China is in a period of economic structural transformation, the supervision mechanism of public opinion is not perfect, and factors such as environmental regulation affect the governance effect of media public opinion to some extent. This paper discussed the relationship between environmental regulation and environmental public opinion pressure, and confirmed that environmental regulation had a moderating effect between environmental public opinion pressure and enterprise green innovation behavior, and found that there were differences in the moderating effects of different types of environmental regulation. The research results can further confirm the “Porter Hypothesis,” deepening the application of institutional theory and legitimacy theory in enterprise green innovation, and broadening the research boundary of the impact of environmental public opinion pressure on enterprise green innovation behavior.

During the “13th 5-Year Plan” period, China's ecological environment has improved significantly, and the phased goals of the battle against pollution have been successfully completed. However, the pressure on ecological environmental protection has not been fundamentally relieved, especially in key areas and industries, the pollution problem is still prominent. China's 14th 5-Year Plan emphasizes improving the quality and stability of ecosystems, continuously improving environmental quality, and accelerating the green transformation of development methods. Therefore, the research conclusions are helpful to provide reference for the green innovation strategy, organizational decision-making and management practice of government departments and construction enterprises.

It is needed to strengthen the supervision and governance functions of environmental public opinion on enterprise green innovation. The government should make full use of the power of media public opinion to strengthen the supervision and governance of enterprise environmental behavior. At the same time, the government should also continuously improve the participation mechanism of the public, consumers and other stakeholders in environmental supervision, and enriching the means of public opinion supervision. Enterprises should turn the pressure of environmental public opinion into the driving force of green innovation, to enhance the competitiveness and sustainable development ability through green innovation, and improve the economic and environmental benefits of enterprises. Material suppliers, investors, consumers and other stakeholders should be good at using media and public opinion to safeguard their own legitimate interests and social fairness and justice, and trigger enterprises to carry out green procurement, green design, green production and operations.

It is important to pay attention to the influence of leaders on enterprise green innovation decision-making. The government should attach importance to the role of leaders in green innovation strategies, strengthen environmental policy training for enterprises leaders, and enhance their psychological identity and subjective desire for environmental protection. At the same time, implementing the dual policy of incentive guidance and punishment intervention, and incorporating the green innovation behavior of enterprise into the reputation evaluation of professional managers are necessary. In the selection and appointment of leaders, enterprises should pay attention to the matching of leaders' values and core social values to strengthen the cultivation of leaders' environmental awareness and appeal of green innovation. Leaders themselves should enhance the sensitivity of environmental policies, deepen the understanding of environmental protection and green innovation, and implement green innovation.

Combining mandatory environmental regulation and incentive environmental regulation to promote green innovation of enterprises is desired. At present, China is in a critical period of economic transformation, and the government governance mechanism is not perfect. There still has a limited effect on improving enterprise environmental governance only relying on incentive environmental regulation. This research finds that mandatory environmental regulation has a stronger positive moderating effect on the relationship between environmental public opinion pressure and enterprise green innovation behavior. Therefore, while continuously improving the government supervision mechanism, the proportion of mandatory environmental regulation can be appropriately increased, so as to better exert the supervision and governance function of media public opinion, promoting enterprise green innovation, and improving the comprehensive management capacity of ecological environment.

### Limitations and Future Prospects

As an exploratory empirical study, this research has some limitations. On the one hand, the construction industry is taken as the empirical object, whether the conclusions of this paper have general guiding significance and suitable for other industries needs further exploration. On the other hand, in addition to leaders' environmental intention and environmental regulation, we have not conducted an in-depth empirical test with other possible factors. Therefore, the future research should expand the scope of the empirical research and enhance the general guiding significance of the research conclusions. Researchers should further study the influence mechanism of environmental public opinion pressure on enterprise green innovation behavior, especially the influence mechanism of environmental public opinion pressure on leaders' environmental intention. Researchers should comparatively analyze the impact of environmental public opinion pressure on enterprise green innovation behavior under different economic system conditions, and further reveal the paths that environmental public opinion pressure affects enterprise green innovation behavior.

## Data Availability Statement

The raw data supporting the conclusions of this article will be made available by the authors, without undue reservation.

## Ethics Statement

Ethical review and approval was not required for the study on human participants in accordance with the local legislation and institutional requirements. The patients/participants provided their written informed consent to participate in this study.

## Author Contributions

BW, YC, and YA: conceptualization. SH: methodology. FL: software and data curation. SH and TW: resources. BW and TW: writing—original draft preparation. BW and SH: data collection. BW, TW, YC, and YA: writing—review and editing. All authors have read and agreed to the published version of the manuscript.

## Funding

This research was funded by the soft science project of sichuan provincial department of science and technology (2022JDR0229). The article processing costs are funded by Delft University of Technology.

## Conflict of Interest

The authors declare that the research was conducted in the absence of any commercial or financial relationships that could be construed as a potential conflict of interest.

## Publisher's Note

All claims expressed in this article are solely those of the authors and do not necessarily represent those of their affiliated organizations, or those of the publisher, the editors and the reviewers. Any product that may be evaluated in this article, or claim that may be made by its manufacturer, is not guaranteed or endorsed by the publisher.
